# Cycloserine-Induced Insomnia and Psychosis in Multidrug-Resistant Pulmonary Tuberculosis - A Case Report

**DOI:** 10.7759/cureus.32963

**Published:** 2022-12-26

**Authors:** Sankalp Yadav

**Affiliations:** 1 Medicine, Shri Madan Lal Khurana Chest Clinic, Moti Nagar, New Delhi, IND

**Keywords:** adverse drug reaction, psychosis, cycloserine, acid fast bacilli (afb), central nervous system tuberculosis

## Abstract

Tuberculosis (TB) is a highly infectious disease due to *Mycobacterium tuberculosis*. The disease presents as drug-sensitive or drug-resistant TB (DR-TB). DR-TB could be of various types like isoniazid mono-resistant, multidrug-resistant tuberculosis (MDR-TB), MDR-rifampicin mono-resistant (MDR-RR), extensively drug-resistant TB (XDR-TB), or pre-extensively drug-resistant TB (pre-XDR-TB). Management of DR-TB is challenging mainly due to longer treatment duration, high pill burden, and adverse drug reactions (ADR) to the second-line anti-TB drugs. Some of these could be life-threatening and require immediate care. Herein, a case of ADR caused by cycloserine in a pulmonary MDR-TB case is presented. All the antitubercular drugs were put on hold, and the symptoms disappeared, only to reappear with the rechallenge of cycloserine. It is emphasized here that a high degree of suspicion with immediate management is imperative to avoid fatal outcomes.

## Introduction

Tuberculosis (TB) is a bacterial disease caused by *Mycobacterium tuberculosis* and is a substantial contributor of morbidity and mortality in high-burden countries [1]. The disease manifests as drug-sensitive or drug-resistant TB (DR-TB). DR-TB could be isoniazid mono-resistant, multidrug-resistant tuberculosis (MDR-TB), MDR-rifampicin mono-resistant (MDR-RR), extensively drug-resistant TB (XDR-TB), or pre-extensively drug-resistant TB (pre-XDR-TB) [2]. The management of DR-TB is a formidable task. The main contributor to this is the adverse drug reactions (ADR) to the second line of anti-TB drugs [3]. Many second-line anti-TB drugs affect the central nervous system (CNS) [3]. And if not treated, these ADRs could lead to fatal outcomes.

Cycloserine a (D-4-amino-3-isoxazolidine) is an important second-line anti-TB drug which is a broad-spectrum antibiotic produced by *Streptomyces orchidaceus* [4]. It was upgraded to WHO group B anti-TB drugs in 2018 [5]. The use of cycloserine is associated with severe ADRs ranging from depression, anxiety, insomnia, delirium, psychosis, and mania [3]. The incidence of cycloserine-induced psychosis in MDR-TB cases is 12% [6]. Further, a pharmacological safety and surveillance study estimated that the global combined prevalence of ADRs due to cycloserine was 9.0%, and 5.7% for psychiatric disorders [7]. Early identification and prompt management of these ADRs are imperative for the treatment's success. A unique case of ADR caused by cycloserine in a pulmonary MDR-TB case is presented here. Even in high-burden countries, there is a paucity of literature regarding the ADRs of cycloserine. It is emphasized that the management of such cases requires a very high degree of suspicion, so the treating physicians and healthcare workers should always be vigilant towards such ADRs.

## Case presentation

**Figure 1:**
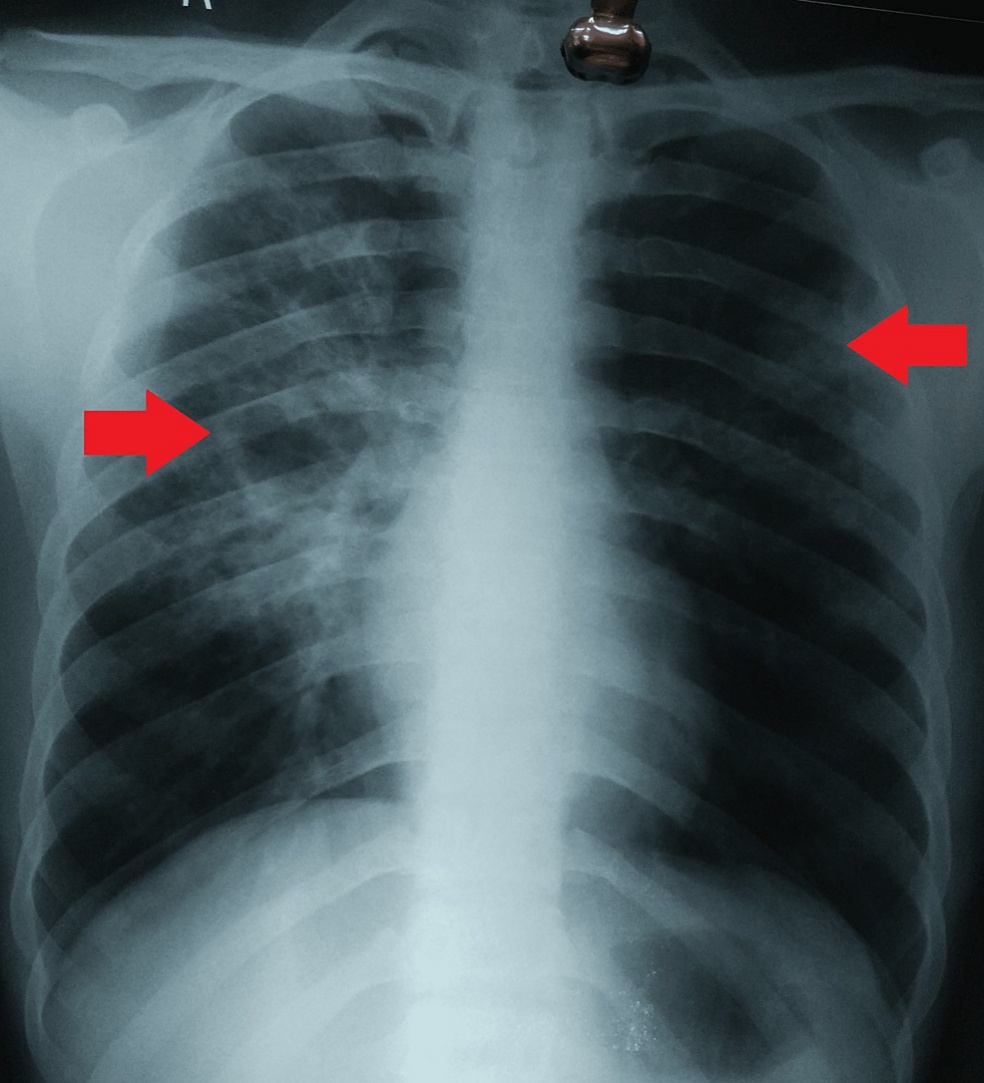
Chest radiograph posteroanterior view suggestive of bilateral lung involvement with patchy opacities and right-sided cavity

A 31-year-old Indian male was brought to the outpatient department (OPD) with complaints of irrelevant talk, violent behavior, loss of sleep, and headache for five days. This patient was on an all-oral longer regimen for treatment of MDR-TB (pulmonary), i.e., in the initial phase for 25 days per the national guidelines. The diagnosis of MDR-TB was based on the reports of sputum microscopy for acid-fast bacilli (AFB), cartridge-based nucleic acid amplification test (CBNAAT), line-probe assay, culture, and drug susceptibility testing, and a chest radiograph (Table 1, Figure 1).

**Table 1:** Diagnostic investigations suggestive of multidrug-resistant pulmonary tuberculosis

Investigations	Results
Sputum microscopy for acid-fast bacilli (AFB)	3+
Cartridge-based nucleic acid amplification test	*Mycobacterium tuberculosis* detected, rifampicin resistance high level
Line-probe assay	Resistance to isoniazid and rifampicin
Culture and drug susceptibility testing	*Mycobacterium tuberculosis* grew, no resistance to second-line anti-tubercular drugs

The patient was well about five days back when he had a throbbing headache which aggravated as the days progressed. The headache was relieved for some time after he took an over-the-counter non-steroidal anti-inflammatory drug (NSAID) (diclofenac). However, it was not associated with visual disturbances, neck stiffness, or new onset of fever. His family members informed that his behavior changed during these five days, and there were episodes during which he started shouting and became restless with irrelevant talking. They also informed that he had not slept for four days. There were no seizures or manic episodes.

There was no major past medical or surgical history except for being treated for drug-sensitive extra-pulmonary TB (left cervical lymphadenitis) one year ago for 168 days per the national guidelines. During that treatment, he received fixed-dose combinations of rifampicin, isoniazid, pyrazinamide, and ethambutol for two months, constituting the intensive phase and followed by rifampicin, ethambutol, and isoniazid for the next four months in the continuation phase. He was a teetotaler with no history of substance abuse. There was no history of any diagnosed psychiatric disorders in the patient and immediate contacts. He also reported having regular sleep without any issues before these five days, and there were no delusions or hallucinations. Besides, there was no history of similar complaints in the past or any conflicts or disputes with family or at work. He was a non-immigrant and there was no history of unemployment, imprisonment, contacts with drug dealers, or commercial sex workers. There was no history of coronavirus disease 2019 (COVID-19), and he was not vaccinated for the same.

General examination revealed a lean man with a temperature of 98.4 degrees Fahrenheit, a pulse of 100 beats/minute, blood pressure of 125/90 mmHg, respiratory rate of 19/minute, and SpO_2_ of 98% on room air. He was well-oriented to time, place, and person. There was no clubbing, cyanosis, icterus, koilonychia, pallor, lymphadenopathy, or edema.

Systemic examination was unremarkable, with no nuchal rigidity and jolt accentuation. Kernig's sign and Brudzinski's sign were negative. On mental status examinations, there was increased psychomotor activity, elevated mood, anxiety, and pressured speech. The Young Mania Rating Scale (YMRS) score was 19, the Naranjo Scale score was 11, and the Brief Psychosis Rating Score (BPRS) score was 36. ADRs were potentially severe on the Hartwig ADR severity assessment scale. The Schumock and Thornton scale for preventability assessment was done, which classified the ADR as definitely preventable (Table 2).

**Table 2:** Detailed mental status examination

Scale	Score	Normal limits
Young Mania Rating Scale	19	≤12 indicates remission
		13-19 = minimal symptoms
		20-25 = mild mania
		26-37 = moderate mania
		38-60 = severe mania
Naranjo scale score	11	-4 to +13
Brief Psychosis Rating score	36	0-126
Hartwig adverse drug reaction (ADR) severity assessment scale	Severe, level 5	Mild = levels 1 and 2
		Moderate = levels 3 and 4
		Severe = levels 5, 6, and 7
The Schumock and Thornton scale	Preventable	Preventable
		Probably preventable
		Non-preventable

Detailed laboratory work-up revealed hemoglobin 10.2 g/dL, total leukocyte count 7.1 × 10^9^/L, differential leukocyte count neutrophils 80, eosinophils 1, monocytes 1, lymphocytes 16, mean corpuscular volume 57 fl, mean corpuscular hemoglobin concentration 21 g/dl, mean corpuscular hemoglobin 20 pg, erythrocyte sedimentation rate 52 mm/h, blood sugar fasting 89 mg/dL, serum creatinine 0.3 mg/dL, normal thyroid profile, serum urea 21 mg/dL, serum bilirubin 1.0 mg/dL, aspartate transferase 41 mg/dL, alanine transferase 39 mg/dL, rapid plasma reagin (RPR) test for HIV- I and II nonreactive, normal vitamin B6 levels, and the thyroid profile and serum electrolytes were within normal limits. His cerebrospinal fluid (CSF) was clear and colorless (total protein: 24 mg/100 mL, CSF glucose: 50 mg/100 mL, CSF cell count: 0 white blood cells, and no red blood cells). CSF fluorescent treponemal antibody absorption (FTA-ABS), the rapid plasma reagin (RPR), and the venereal disease research laboratory (VDRL) tests were negative. A non-contrast computerized tomography (NCCT) of the head was within normal limits.

Based on the chief complaints and clinical examination, a provisional diagnosis of drug-induced psychosis was made after ruling out differential diagnoses like hepatic encephalopathy, meningitis, tuberculoma, neurosyphilis, and spinal arachnoiditis and the patient was referred to the inpatient facility for admission at the nodal DR-TB center. He was on an all-oral longer regimen for 25 days that included tablet bedaquiline 400 mg once a day for two weeks followed by 200 mg alternate day for 22 weeks, with an optimized background regimen consisting of tablet linezolid 600 mg once daily, tablet levofloxacin 1000 mg once daily, tablet clofazimine 100 mg once daily, tablet cycloserine 750 mg once a day, and tablet pyridoxine 100 mg once daily. All these drugs were put on hold. On admission under the team of a psychiatrist and an infectious diseases expert, he was given an injection of lorazepam 2 mg at bedtime, an injection of promethazine 50 mg intramuscular once a day, an injection of haloperidol 5 mg intramuscular twice a day, a tablet nitrazepam 10 mg twice daily, a tablet olanzapine 10 mg twice daily, and tablet thiamine 100 mg three times a day. On the third day post-admission, he showed improvement, with the majority of his symptoms ebbed. His antipsychotic drugs were discontinued from the seventh day after tapering before the rechallenge of MDR-TB drugs.

From the eighth day, a drug rechallenge was done under the guidance of a psychiatrist. On rechallenging the drugs, no ADR was reported except on the administration of cycloserine (after eight hours) when there was a relapse of symptoms with reduced sleep, excessive talk, violent crying, and restlessness. All the drugs were stopped again, and the patient returned to normalcy on the third day. Therefore, a final diagnosis of cycloserine-induced psychosis was made, and his regimen was modified with the omission of cycloserine and the addition of ethionamide as per his weight. After modification in his MDR-TB regimen, there were no episodes of psychosis or insomnia. A detailed mental health examination considering all the scales which were suggestive of psychosis were within the normal limits. Therefore, he was discharged after one week of initiating ethionamide with advice for regular follow-ups (initially every week/as and when required, then every month till the sixth month and quarterly from the seventh month onwards) in the psychiatry OPD and at the nodal DR-TB center for the complete duration of 18 months.

## Discussion

ADRs due to second-line anti-TB drugs are widely reported [3,4,6]. These ADRs are common during the treatment of DR-TB cases [3]. The situation becomes difficult when these ADRs are life-threatening [8]. Regular follow-ups in the OPD and counseling are important parts of the management of DR-TB cases [3]. However, all these are difficult in high-burden settings with a very high load of patients.

Drugs like isoniazid, ethambutol, cycloserine, and fluoroquinolones are known to cause psychosis [3]. Based on the published data, it is evident that cycloserine has higher tenacity to cause psychiatric and central nervous system (CNS)-related ADRs [9]. In the present case, an Indian male came to the OPD, and after all clinical and diagnostic interventions, the psychosis was attributed to cycloserine, and the drug was withdrawn. The resolution of the psychosis after drug withdrawal was clear evidence of drug-induced psychosis. Moreover, no other antitubercular drug was responsible, as the symptoms of psychosis were not seen once the drugs were restarted. It should be emphasized here that a delay in reporting such difficult cases could result in adverse outcomes [8]. Reports of cycloserine-induced psychosis resulting in suicides are available in the literature [8].

The exact mechanism of cycloserine resulting in psychosis is not known, but few studies support the role of cycloserine as a partial agonist of N-methyl-D-aspartate (NMDA) receptors binding to glycine sites [10,11]. Further, at high doses, it can act as an NMDA receptor antagonist [12,13], which could result in generating or exacerbating psychotic symptoms [10,14]. However, another study could not associate the dose-dependent effects of cycloserine resulting in psychosis [15]. 

Few cases and case series are available in the literature due to cycloserine-induced psychosis. A case similar to this case was published by Tandon et al. in 2015, where the patient had aggressive and violent behavior, anxiety, insomnia, and restlessness along with severe hepatic dysfunction [4]. However, the present case differs from their case in normal liver function. Another similar case by Sharma et al. 2014, reported psychosis, delusions, and hallucinations, but the present case differs from their case in the presence of headaches, with the absence of delusions and hallucinations [16].

Cycloserine is an essential part of DR-TB regimens, but it could result in serious ADRs. It is imperative to have a psychiatric evaluation done for all cases before starting this drug. Per the national guidelines in the pretreatment evaluation of DR-TB cases like MDR-TB, psychiatric evaluation is always done before initiating the drugs [17]. This patient underwent a psychiatric evaluation before his treatment initiation but ended up with cycloserine-induced psychosis, which stresses the need for a regular psychiatric assessment during the treatment to prevent any fatal episodes.

## Conclusions

A unique case of ADR of cycloserine in a young Indian male is presented. Prompt management with regular follow-ups and counseling helped this case to be managed with no fatalities. There is evidence of cycloserine-induced psychosis resulting in suicides in cases on treatment for MDR-TB. The most important message from this case is that delays in presentations and management could lead to unfavorable outcomes, and thus regular follow-ups of patients in the OPD per the national guidelines and counseling of their family members for prompt reporting of any abnormal feelings or behavior is imperative. Furthermore, efforts to keep the patients in touch with the health facilities, especially when the visits to the OPD are difficult, are very important. Up-to-date training of healthcare workers is also very important for timely detection and early management.
